# Dark clouds of contrast

**DOI:** 10.1007/s12471-019-1258-x

**Published:** 2019-05-14

**Authors:** M. Boulaksil, J. E. M. Mellema, T. J. F. ten Cate

**Affiliations:** 1 Department of Cardiology, Radboud University Medical Center, Nijmegen, The Netherlands; 2 Department of Cardiology, Slingeland Hospital, Doetinchem, The Netherlands

## Answer

**Fig. 1:**
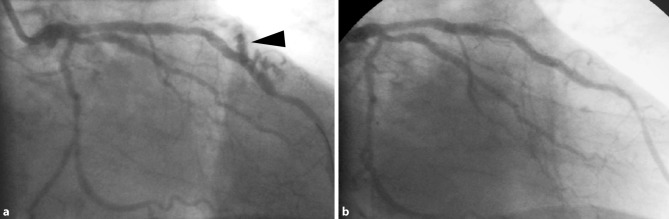
**a** Still image of right anterior oblique (RAO) view of coronary angiogram of our patient. The arrow head shows a chimney of contrast indicative of contrast extravasation caused by coronary perforation. See also the online video (online supplement). **b** Still image (RAO view) showing the final result after covered stent implantation

Notably, the two-vessel disease consisted of an obtuse marginal branch and two lesions in the left anterior descending (LAD) artery (Fig. 1a and online video). We decided on percutaneous coronary intervention (PCI) of the LAD. However, the distal lesion was partially localised in an intramural segment of the LAD (so called ‘bridging’). After direct stenting of the proximal LAD lesion, the distal lesion was predilated and a drug-eluting stent was implanted. Thereafter, ‘chimneys of contrast’ were perceived running into the myocardium and pericardium (arrow head in Fig. 1a and online video). These chimneys are extravasations of contrast caused by coronary perforation.

Coronary perforation in PCI occurs in 0.2–0.3% of cases, depending on PCI complexity, and is associated with increased mortality risk [1, 2]. For grading of coronary perforation, the Ellis classification is most commonly used [3]. Implantation of a covered stent is one of the therapeutic options, though with an increased risk of in-stent thrombosis and restenosis [2].

In our patient, a covered stent was subsequently implanted to seal the perforation (Fig. 1b). Although the clinical course was complicated by recurrent episodes of mild pericarditis, our patient could be discharged five days after the PCI. This case emphasises that stenting of intra-mural coronary arteries, even when partially intramural, should be discouraged.

## Caption Electronic Supplementary Material

**Video 1. **Coronary angiography (RAO view) of our patient
